# Superior patient‐reported clinical outcomes after patient‐individual total knee arthroplasty compared with off‐the‐shelf robotic‐assisted total knee arthroplasty in valgus knee phenotypes

**DOI:** 10.1002/ksa.70399

**Published:** 2026-04-16

**Authors:** Lars‐Rene Tuecking, Peter Savov, Mara Hold, Ricarda Stauss, Henning Windhagen, Max Ettinger

**Affiliations:** ^1^ Hannover Medical School Clinic of Orthopaedic Surgery in Diakovere Annastift Hannover Germany; ^2^ Department of Orthopaedic and Trauma Surgery, Pius Hospital Carl von Ossietzky University of Oldenburg Oldenburg Germany

**Keywords:** CPAK, patient‐individual, robotic‐assisted, total knee arthroplasty, valgus

## Abstract

**Purpose:**

To compare early patient‐reported clinical outcomes of patient‐individual total knee arthroplasty (TKA) with individualized trochlear design versus robotic‐assisted TKA using off‐the‐shelf implants in patients with valgus knee phenotypes and pronounced femoral valgus (lateral distal femoral angle [LDFA] < 85°) at 3 and 12 months postoperatively in a prospective matched case–control study.

**Methods:**

This prospective matched case–control study based on predefined anatomical criteria included 42 patients who underwent primary TKA between October 2021 and October 2023. Twenty‐one patients received patient‐individual posterior‐stabilized implants. Twenty‐one matched controls underwent robotic‐assisted TKA with off‐the‐shelf implants. All procedures followed restricted kinematic alignment (KA) principles with valgus undercorrection. Primary outcomes were patient‐reported scores (Forgotten Joint Score [FJS], Kujala Score, Oxford Knee Score [OKS] and the Western Ontario and McMaster Universities Arthritis Index [WOMAC]), assessed preoperatively and at 3 and 12 months.

**Results:**

Both groups demonstrated comparable preoperative clinical status and comparable degrees of preoperative valgus deformity, with no significant differences in hip–knee–ankle angle (HKA, 185.6 ± 6.3° vs. 188.8 ± 4.1°; *p* = 0.060) or mechanical LDFA (84.0 ± 0.9° vs. 83.7 ± 1.2°; *p* = 0.261). At 3 and 12 months, patient‐individual total knee arthroplasty (pTKA) demonstrated significantly better scores across all patient‐reported outcome measures (PROMs) (*p* < 0.05). The largest difference was observed in the Kujala score at 12 months (79.5 ± 12.8 vs. 59.7 ± 19.0, *p* = 0.001). FJS improved more rapidly in the pTKA group (from 9.0 to 49.1 at 3 months, and 52.5 at 12 months) compared to robotic‐assisted total knee arthroplasty (rTKA) (26.9 and 36.0, respectively). One revision for suspected infection occurred in the pTKA cohort; none were reported after rTKA.

**Conclusions:**

Patient‐individual TKA with individualized trochlear design achieved superior early functional outcomes compared to robotic‐assisted TKA with standard implants in severe valgus morphotypes. Individualized trochlear orientation may improve patellofemoral mechanics and facilitate faster recovery in patients with severe femoral valgus.

**Level of Evidence:**

Level III.

**Abbreviations**
aHKA: arithmetic hip–knee–ankle angle
BCS: bi‐cruciate stabilized
CORI: CORI robotic system
CPAK: Coronal Plane Alignment of the Knee
CR: cruciate‐retaining
CT: computed tomography
EZR: Easy R
FJS: Forgotten Joint Score
KA: kinematic alignment
LDFA: lateral distal femoral angle
MA: mechanical alignment
OKS: Oxford Knee Score
PROMs: patient‐reported outcome measures
PS: posterior‐stabilized
PSI: patient‐specific instrumentation
pTKA: patient‐individual total knee arthroplasty
R: R (programming language)
rKA: restricted kinematic alignment
rTKA: robotic‐assisted total knee arthroplasty
TKA: total knee arthroplasty
WOMAC: Western Ontario and McMaster Universities Arthritis Index

## INTRODUCTION

To address a remaining dissatisfaction rate of 10%–20% in primary total knee arthroplasty (TKA) [4], surgical strategies have increasingly focused on improving functional outcomes by better restoring native knee anatomy and joint mechanics [14]. More advanced methods have introduced more refined alignment philosophies that attempt to reconstruct the patient's native alignment and joint line orientation [16]. However, even personalized alignment strategies using off‐the‐shelf implants might not always show consistent results across all anatomical phenotypes [7, 19].

Particularly, valgus knee morphotypes such as Coronal Plane Alignment of the Knee (CPAK) Types III and VI have reported inferior outcomes following personalized alignment philosophies [7]. These patients typically have increased lateral distal femoral angles (LDFAs), necessitating valgus orientation of the femoral component in these alignment techniques. This alignment may produce a medially displaced trochlear groove compared with the quadriceps vector, which might lead to impaired patellar tracking or lateral overcompression—both of which can potentially play a role in leading to inferior clinical results [8]. One potential disadvantage of off‐the‐shelf standard implants is their fixed trochlear orientation, especially in the case of high femoral valgus [11]. Patient‐individual implants with personalized trochlear design offer a potential alternative by more closely approximating native anatomy, trochlear orientation and potentially evading patellofemoral complications [2, 12].

The purpose of this prospective comparative study was to evaluate whether patient‐individual TKA with an individualized trochlear design results in superior patient‐reported clinical outcomes compared with robotic‐assisted TKA using off‐the‐shelf implants in patients with valgus morphotypes and high femoral valgus (LDFA < 85°) at early postoperative state. We hypothesized that patient‐individual implants, by allowing customization of trochlear orientation, would lead to improved patellofemoral‐related outcomes and overall patient outcome scores compared with standard implant designs in this challenging valgus population.

## METHODS

In this prospective, comparative cohort study, a total of 42 patients who underwent TKA between October 2021 and October 2023 were included. During this period, a consecutive case series of 21 patients received patient‐individual implants (Origin PS; Symbios, Switzerland), selected according to predefined inclusion criteria. All patients presented with valgus morphotypes corresponding to CPAK Types III or VI. Additional inclusion criteria included a LDFA of less than 85°, and a constitutional valgus leg alignment, defined by an arithmetic hip–knee–ankle angle (aHKA) greater than 5° (Table 1). A partial correction within the physical examination of the valgus deformity was required in all cases.

**Table 1:** Inclusion and exclusion criteria.

Inclusion criteria	Exclusion criteria
aHKA > 5°	Post‐traumatic osteoarthritis
CPAK Type III or VI	Fixed valgus limb alignment
LDFA < 85°	Severe medial laxity with possible indication for constrained implant designs
Partial reducibility of valgus limb axis	
Age > 18 years	

*Note*: Overview of the predefined eligibility criteria used for patient selection in the study, including inclusion criteria and exclusion criteria applied during recruitment.

Abbreviations: aHKA, arithmetic hip–knee–ankle angle; CPAK, coronal plane alignment of the knee; LDFA, lateral distal femoral angle.

The control group consisted of the first 21 consecutive patients identified from the institutional prospective knee arthroplasty registry who received robotic‐assisted off‐the‐shelf implants (Journey II BCS; Smith & Nephew, USA) during the same study period. Matching was based exclusively on predefined anatomical and radiographic criteria, including CPAK Type (III/VI), LDFA < 85°, constitutional valgus alignment (aHKA > 5°) and partial reducibility of the valgus deformity. Treatment allocation was not randomized. During a defined implementation interval of patient‐individual TKA at our institution, all consecutive patients meeting the predefined anatomical inclusion criteria received the individualized implant. Outside this defined interval, patients fulfilling identical criteria were treated with robotic‐assisted off‐the‐shelf TKA according to standard institutional practice. Allocation was therefore based on institutional treatment pathways and implant availability rather than patient‐specific prognostic factors. To further minimize variability, all procedures in both groups were performed by a single, experienced surgeon. Perioperative management did not differ in between groups, the same perioperative management protocol was used for all patients included in this study.

Ethical approval was obtained from the local ethics committee (Reference No.: 8808_BO_K_2019), and written informed consent was obtained from all participants prior to study inclusion.

All procedures were performed using a valgus undercorrection strategy consistent with the principles of restricted kinematic alignment (rKA) without use of patellar resurfacing. In the patient‐individual implant group, patient‐specific instrumentation (PSI) based on preoperative computed tomography (CT) was used. In the control group, off‐the‐shelf implants were implanted using an imageless robotic‐assisted system (CORI, Smith&Nephew, USA). Both techniques aimed to achieve undercorrection of valgus alignment, targeting a residual mechanical axis of ≤3° valgus and a maximum component inclination of 5° for both femoral and tibial cuts.

In robotic‐assisted procedures, additional efforts were made to reconstruct the native trochlear orientation within the permitted alignment boundaries. For patient‐individual implants, the trochlear groove was individually planned to match the patient's anatomy, allowing for decoupling of the tibiofemoral and patellofemoral joints. In cases of lateral compartment stiffness, soft‐tissue balancing was performed in a stepwise manner following established protocols as previously described [18].

The primary outcome was clinical performance assessed via validated patient‐reported outcome measures (PROMs), including the Forgotten Joint Score (FJS), Kujala Score, Oxford Knee Score (OKS) and the Western Ontario and McMaster Universities Arthritis Index (WOMAC). PROMs were recorded preoperatively and at two postoperative time points: 3 and 12 months. Additionally, revision events, including time point and reason for revision, were documented at final follow‐up.

Standardized, full‐length, weight‐bearing anteroposterior radiographs of the lower extremity were obtained preoperatively and postoperatively in all patients. Radiographic measurements included the HKA, aHKA, mechanical lateral distal femoral angle (mLDFA), mechanical medial proximal tibial angle (mMPTA) and joint line obliquity (JLO). All measurements were performed according to established definitions using calibrated digital imaging software (Carestream; Carestream Health Inc., USA).

Statistical analysis was conducted using R software with the EZR (Easy R) plugin. Demographic variables and baseline PROM scores were compared prior to outcome analysis to assess initial group comparability. Continuous variables were summarized using means and standard deviations, while categorical variables were reported as frequencies and percentages. Between‐group comparisons were performed using unpaired, nonparametric *t* tests and Wilcoxon rank‐sum tests for nominal data. A *p* value < 0.05 was considered statistically significant. Between‐group effect sizes were calculated as Hedges' *g* with 95% confidence intervals based on group means and standard deviations. Post hoc (observed) power was estimated using a two‐sided *α* = 0.05 for independent samples; effect sizes and confidence intervals were considered the primary measures of clinical and statistical relevance.

## RESULTS

### Group comparability

Demographic characteristics showed good comparability between the patient‐individual and the robotic‐assisted control group (Table 2). Radiographic analysis confirmed similar valgus alignment patterns across groups, with no significant differences in LDFA and aHKA (Table 2). Preoperative PROMs were comparable between both cohorts (Table 3). Postoperatively, radiological outcome parameters were comparable between both groups, except for differences for postoperative HKA (Table 2).

**Table 2:** Demographic data of groups.

	rTKA		pTKA		*p* value
Age (years)	65.4 ± 8.9 (48.0–81.0)		69.3 ± 9.7 (55.0–90.0)		0.226
BMI (kg/m^2^)	32.3 ± 5.4 (23.7–42.9)		30.1 ± 4.9 (21.6–38.0)		0.159
			
Gender (female, in %)	16/21 (76.2%)		17/21 (80.9%)		1.000
CPAK			
Type III	20 (95.2%)		20 (95.2%)		1.000
Type VI	1 (4.8%)		1 (4.8%)		
HKA in °					
Preoperative	185.58 ± 6.3 (177.2–196.2)		188.8 ± 4.1 (183.3–199.4)		0.060
Postoperative	181.5 ± 2.9 (179.3–184.6)		183.3 ± 1.7 (180.3–185.7)		**0.004**
aHKA in °					
Preoperative	5.6 ± 2.1 (2.2–9.2)		6.7 ± 2.6 (2.3–12.1)		0.143
Postoperative	3.9 ± 2.6 (2.1–4.9)		3.9 ± 1.4 (2.5–4.7)		0.989
mMPTA in °					
Preoperative	89.6 ± 2.0 (85.3–92.9)		90.4 ± 2.0 (87.0–94.3)		0.195
Postoperative	90.3 ± 1.4 (87.9–91.8)		90.8 ± 1.3 (88.2–92.2)		0.314
mLDFA in °					
Preoperative	84.0 ± 0.9 (80.5–84.9)		83.7 ± 1.2 (79.8–84.9)		0.261
Postoperative	86.3 ± 1.8 (84.4–88.2)		86.9 ± 1.1 (84.8–87.3)		0.299
JLO in °					
Preoperative	173.5 ± 2.0 (168.4–177.1)		173.8 ± 2.0 (171.2–178.5)		0.563
Postoperative	176.6 ± 1.9 (174.8–180.2)		177.7 ± 1.88 (175.4–181.4)		0.165

*Note*: Mean ± standard deviation (Min–Max). Baseline demographic characteristics and radiographic alignment parameters of patients in the rTKA and pTKA groups. Data are presented as mean ± standard deviation with range (minimum–maximum) or counts with percentages, as appropriate. Bold value indicates statistically significant.

Abbreviations: aHKA, arithmetic HKA; BMI, body mass index; CPAK, Coronal Plane Alignment of the Knee classification; HKA, hip–knee–ankle angle; JLO, joint line obliquity; mLDFA, mechanical lateral distal femoral angle; mMPTA, mechanical medial proximal tibia angle; pTKA, patient‐individual total knee arthroplasty; rTKA, robotic‐assisted total knee arthroplasty.

**Table 3:** Patient‐reported outcome measurements.

	rTKA	pTKA	*p* value
Forgotten Joint Score			
Preoperative	9.0 ± 7.6	9.1 ± 7.9	0.936
3 months	26.9 ± 21.5	49.1 ± 24.8	**0.007**
12 months	36.0 ± 23.0	52.5 ± 21.1	**0.018**
Kujala Score			
Preoperative	43.3 ± 14.6	45.4 ± 9.7	0.440
3 months	52.2 ± 15.5	67.3 ± 19.0	**0.016**
12 months	59.7 ± 19.0	79.5 ± 12.8	**0.001**
Oxford Knee Score			
Preoperative	21.0 ± 7.2	20.6 ± 5.4	1.000
3 months	29.6 ± 8.4	35.7 ± 6.3	**0.014**
12 months	34.2 ± 9.1	40.5 ± 5.2	**0.016**
WOMAC Function Score			
Preoperative	56.9 ± 19.6	54.6 ± 15.2	0.953
3 months	29.8 ± 16.6	11.3 ± 10.5	**0.001**
12 months	21.3 ± 19.2	10.7 ± 12.2	**0.056**
WOMAC Pain Score			
Preoperative	57.5 ± 25.5	62.0 ± 13.8	0.972
3 months	35.5 ± 18.9	16.5 ± 13.0	**0.002**
12 months	26.9 ± 17.2	9.6 ± 7.5	**0.001**
WOMAC Stiffness Score			
Preoperative	57.2 ± 21.7	58.5 ± 16.1	0.991
3 months	31.1 ± 19.3	15.5 ± 10.2	**0.006**
12 months	24.4 ± 18.6	14.0 ± 14.7	**0.065**
WOMAC Total Score			
Preoperative	57.2 ± 20.8	58.5 ± 13.5	0.944
3 months	32.2 ± 16.5	14.5 ± 9.8	**0.001**
12 months	24.3 ± 17.4	11.5 ± 9.3	**0.019**

*Note*: Mean ± standard deviation. Comparison of patient‐reported outcome measures between the rTKA and pTKA groups at the preoperative baseline, 3 months and 12 months postoperatively. Values are presented as mean ± standard deviation with corresponding *p* values. Bold values indicate statistically significant.

Abbreviations: pTKA, patient‐individual total knee arthroplasty; rTKA, robotic‐assisted total knee arthroplasty; WOMAC, Western Ontario and McMaster Universities Arthritis Index.

### Patient‐reported outcomes

At both 3‐ and 12‐month follow‐up, patient‐individual total knee arthroplasty (pTKA) reported significantly better patient‐reported outcomes compared to robotic‐assisted TKA (rTKA) as shown in Table 3. The pTKA group performed superior in Kujal Score (Figure 1), FJS (Figure 2), WOMAC Score and OKS at all postoperative time points (*p* < 0.05 for all scores). The most pronounced difference was observed in the Kujala Score at 12 months, with pTKA patients achieving a mean score of 79.5 ± 12.8 compared to 59.7 ± 19.0 in the rTKA group (*p* = 0.001), indicating enhanced functional recovery and patellofemoral joint‐related satisfaction in the patient‐individual cohort.

**Figure 1:**
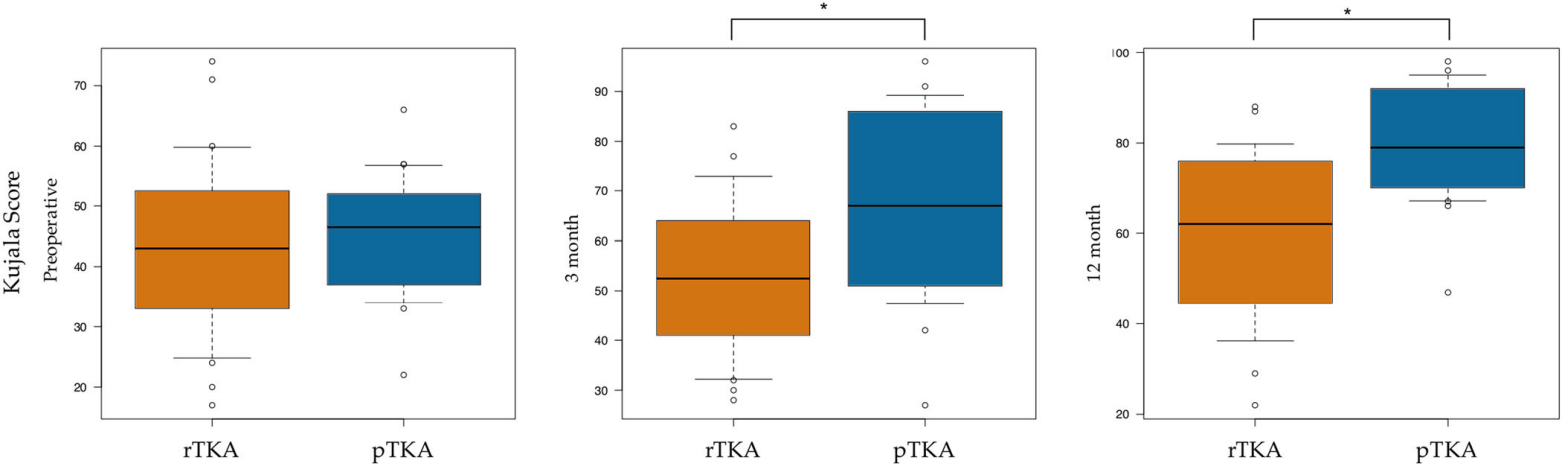
Comparison of group‐specific perioperative Kujala Scores. Box plots illustrating the distribution of Kujala scores in the robotic‐assisted total knee arthroplasty (rTKA, orange) and patient‐individual total knee arthroplasty (pTKA, blue) groups at the preoperative baseline, 3‐month follow‐up and 12‐month follow‐up. Boxes represent the interquartile range, the horizontal line within the box indicates the mean, whiskers indicate the range, and dots represent outliers. Asterisks indicate statistically significant differences (*p* < 0.05) between groups.

**Figure 2:**
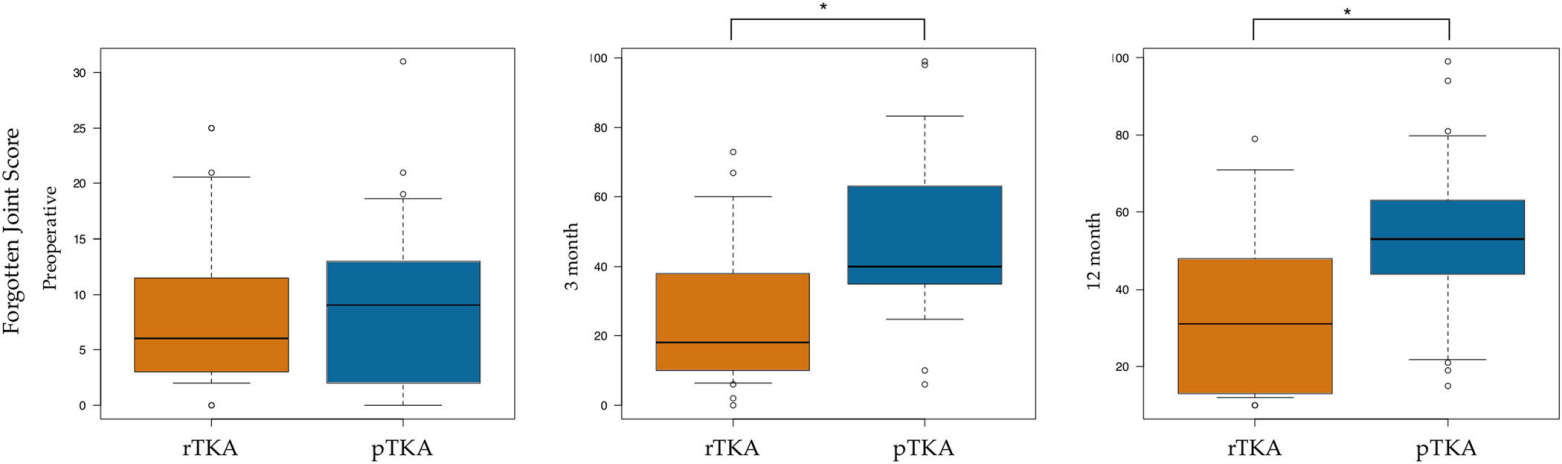
Comparison of group‐specific perioperative Forgotten Joint Scores (FJS). Box plots illustrating the distribution of FJS in the robotic‐assisted total knee arthroplasty (rTKA, orange) and patient‐individual total knee arthroplasty (pTKA, blue) groups at the preoperative baseline, 3‐month follow‐up and 12‐month follow‐up. Boxes represent the interquartile range, the horizontal line within the box indicates the mean, whiskers indicate the range and dots represent outliers. Asterisks indicate statistically significant differences (*p* < 0.05) between groups.

Inter‐group comparisons revealed both groups to have postoperative improvement over short‐term follow‐up. The pTKA group exhibited a rapid early improvement across all PROMs, particularly in the first 3 months. Notably, the FJS increased from 9.0 ± 7.6 preoperatively to 49.1 ± 24.8 at 3 months and further to 52.5 ± 21.1 at 12 months. In contrast, the rTKA group showed a more gradual increase, reaching only 26.9 ± 21.5 at 3 months and 36.0 ± 23.0 at 12 months.

At 12 months, patient‐individual TKA showed a clinically relevant superiority over the reference implant across multiple PROMs. In addition to the Kujala score (mean difference +19.8 points; 95% CI +9.7 to +29.9; Hedges' *g* = 1.20), for which the post hoc (observed) power was high (~0.97), meaningful benefits were observed for the OKS (mean difference +6.3; 95% CI +1.6 to +11.0; *g* = 0.83) and the FJS (mean difference +16.5; 95% CI +2.7 to +30.3), both indicating moderate‐to‐large standardized effects. Furthermore, WOMAC pain scores were substantially lower in the patient‐individual TKA group (mean difference −17.3; 95% CI −25.7 to −8.9; *g* = −1.28). Collectively, these effect magnitudes and confidence intervals exceed established thresholds for clinical importance, underscoring a consistent and patient‐perceived benefit rather than isolated statistical significance.

Within the 12‐month follow‐up period, one revision procedure was required in the pTKA group due to suspected periprosthetic joint infection, managed by isolated polyethylene insert exchange. No revision surgeries occurred in the rTKA cohort.

## DISCUSSION

The main finding of this study indicates that pTKA utilizing an individualized trochlear design resulted in superior early functional and self‐reported clinical outcome compared to rTKA with standard off‐the‐shelf implants in patients with valgus morphotypes (CPAK Type III and VI) and high femoral valgus using a rKA principle. Particularly, patellofemoral joint function scores were markedly improved during the early postoperative phase in pTKA, which may be attributed to the individualized orientation of the prosthetic trochlea in these cases.

Analogous to the improved clinical outcomes observed with KA compared to mechanical alignment (MA) in varus morphotypes [5], superior results have also been demonstrated for KA in valgus knee anatomies when compared to MA TKA [1]. KA is observed to enhance joint biomechanics by increasing stability through preserved medial collateral ligament isometry [20], avoiding increased constraint levels [1, 18] and improving patellar tracking and patella loading [10, 15]. Despite possible advantages of KA, limitations arise when using standard off‐the‐shelf femoral components, which are not optimized for valgus component alignment. Maintaining severe femoral valgus orientation can lead to medialization of the prosthetic trochlear groove to the quadriceps vector in off‐the‐shelf implants due to a fixed trochlear orientation in those femoral components [8]. This mismatch contributes to lateral patellar overstuffing and impaired patellar tracking, both of which are associated with reduced patient satisfaction and delayed early recovery [7].

Independent of the alignment strategy used, there is substantial variation in distal femoral anatomy between individuals, including marked differences in trochlear orientation relative to the distal femoral joint line and the posterior condylar axis [6]. Large CT‐based studies have shown that the shape and orientation of the native trochlea vary widely among patients and are often not aligned with the standardized reference axes used in conventional implant designs [13]. Several studies have therefore described a mismatch between native trochlear orientation and the fixed trochlear geometry of off‐the‐shelf femoral components, regardless of the alignment philosophy applied [17]. Such a mismatch may negatively affect patellofemoral mechanics, particularly when standard implants are used in anatomically complex knees. The use of individualized trochlear orientation in the pTKA femoral components likely addressed these issues in patients with valgus phenotypes with pronounced femoral valgus, which may explain the significantly higher Kujala scores observed in this study. These findings are consistent with prior reports that emphasize the importance of restoring native trochlear anatomy to optimize clinical outcomes [9].

In addition to superior absolute outcomes, the pTKA group exhibited a more rapid improvement in PROMs during the early postoperative period. This was most notable in the FJS, which increased from 9.0 preoperatively to 49.1 at 3 months, compared to a more modest increase in the rTKA group. This pattern suggests that personalized implant geometry may facilitate a faster recovery with reduced joint awareness, possibly due to reduced soft‐tissue irritation or better restoration of native joint mechanics. Similar early benefits have been described in kinematically aligned or functionally aligned TKA cohorts [21, 22], further supporting the potential role of personalization in enhancing early recovery. These results underscore the potential advantages of patient‐individual implant systems in addressing the complex anatomy of valgus knees, particularly in cases of high femoral valgus (LDFA < 85°) configurations. The ability to decouple tibiofemoral and patellofemoral design—as achieved through patient‐individualized implants—may offer distinct biomechanical and functional benefits in these cases [3]. This supports a tailored approach to TKA planning that extends beyond alignment philosophy alone and incorporates anatomical customization.

Despite its strengths, including the prospective design, strict inclusion criteria and consistent surgical technique, this study has several limitations. First, the relatively small sample size and 12‐month follow‐up preclude conclusions regarding long‐term implant survival and durability. Nevertheless, the consistently moderate‐to‐large effect sizes with corresponding confidence intervals—particularly for patellofemoral‐related outcomes—suggest that the observed differences are unlikely to be explained by random variation alone. Second, the study design inherently combines several factors that may act as potential confounders, including patient‐individual versus off‐the‐shelf implant design and PSI versus robotic assistance. Although all procedures followed predefined rKA principles, minor intraoperative adjustments within the permitted alignment boundaries may have resembled elements of functional adaptation. This represents a potential source of residual confounding. In addition, despite standardized soft‐tissue balancing protocols, subtle differences in intraoperative soft‐tissue management between groups cannot be entirely excluded. Furthermore, although patients were matched based on predefined valgus phenotype criteria, preoperative and postoperative hip–knee–ankle (HKA) angles were not fully comparable between groups. The patient‐individual TKA group demonstrated slightly more valgus postoperative HKA values and greater deviation from a neutral limb axis, yet achieved superior clinical outcomes. However, preoperative joint laxity and soft‐tissue characteristics—assessed clinically but not quantitatively measured—may have influenced postoperative alignment and early functional recovery. These factors should therefore be considered when interpreting the results. The single‐center design and use of a specific implant platform limit generalizability. Finally, as this study represents a prospective matched case–control design, residual confounding cannot be fully excluded and definitive causal conclusions require confirmation in adequately powered prospective randomized controlled trials.

## CONCLUSION

In conclusion, patient‐individual TKA appears to offer meaningful improvements in early functional outcomes in valgus morphotype with high femoral valgus compared to robotic‐assisted TKA with off‐the‐shelf implants. These findings support the hypothesis that anatomical customization, particularly of the trochlear groove, may play a critical role in optimizing outcomes in complex knee morphotypes.

## AUTHOR CONTRIBUTIONS

*Conceptualization*: Lars‐Rene Tuecking, Max Ettinger and Peter Savov. *Data curation*: Lars‐Rene Tuecking, Peter Savov, Mara Hold and Ricarda Strauss. *Formal analysis*: Lars‐Rene Tuecking and Peter Savov. *Investigation*: Lars‐Rene Tuecking, Mara Hold, Ricarda Strauss, Max Ettinger and Peter Savov. *Methodology*: Lars‐Rene Tuecking, Peter Savov, Henning Windhagen and Max Ettinger. *Project administration*: Max Ettinger, Lars‐Rene Tuecking and Henning Windhagen. *Supervision*: Max Ettinger and Henning Windhagen. *Visualization*: Lars‐Rene Tuecking and Peter Savov. *Writing—original draft*: Lars‐Rene Tuecking, Max Ettinger. *Writing—review and editing*: Lars‐Rene Tuecking, Peter Savov, Mara Hold, Ricarda Strauss, Max Ettinger and Henning Windhagen.

## CONFLICT OF INTEREST STATEMENT

Max Ettinger, Lars‐Rene Tuecking and Peter Savov have been paid educational consultancy fees from Symbios, Switzerland and Smith & Nephew, USA. The remaining authors declare no conflicts of interest.

## ETHICS STATEMENT

This study was approved by the local Ethics Committee (EK‐Nr. EK 8808_BO_K_2019). Written informed consent was received from every included patient.

## Data Availability

The datasets generated and/or analyzed during the current study are available from the corresponding author on reasonable request.

## References

[1] Bar‐Ziv Y , Beit Ner E , Lamykin K , Essa A , Gilat R , Livshits G , et al. Minimum 2‐year radiographic and clinical outcomes of kinematic alignment total knee arthroplasty in the valgus knee. J Pers Med. 2022;12(7):1164. 10.3390/jpm12071164 PMC9318663 35887663

[2] Beel W , Sappey‐Marinier E , Latifi R , Aït‐Si‐Selmi T , Bonnin MP . Individualised compared to off‐the‐shelf total knee arthroplasty results in lower and less variable patellar tilt. Knee Surg Sports Traumatol Arthrosc. 2024;32(12):3163–3173. 10.1002/ksa.12234 38864165

[3] Bonnin M , Saffarini M , Lustig S , Hirschmann MT . Decoupling the trochlea from the condyles in total knee arthroplasty: the end of a curse? Knee Surg Sports Traumatol Arthrosc. 2024;32(7):1645–1649. 10.1002/ksa.12267 38769816

[4] DeFrance MJ , Scuderi GR . Are 20% of patients actually dissatisfied following total knee arthroplasty? A systematic review of the literature. J Arthroplasty. 2023;38(3):594–599. 10.1016/j.arth.2022.10.011 36252743

[5] Ettinger M , Tuecking LR , Savov P , Windhagen H . Higher satisfaction and function scores in restricted kinematic alignment versus mechanical alignment with medial pivot design total knee arthroplasty: a prospective randomised controlled trial. Knee Surg Sports Traumatol Arthrosc. 2024;32(5):1275–1286. 10.1002/ksa.12143 38501253

[6] Hazratwala K , O'Callaghan WB , Dhariwal S , Wilkinson MPR ; 360 Knee Group. Wide variation in tibial slopes and trochlear angles in the arthritic knee: a CT evaluation of 4116 pre‐operative knees. Knee Surg Sports Traumatol Arthrosc. 2022;30(9):3049–3060. 10.1007/s00167-021-06725-2 34487188

[7] Howell SM , Gill M , Shelton TJ , Nedopil AJ . Reoperations are few and confined to the most valgus phenotypes 4 years after unrestricted calipered kinematically aligned TKA. Knee Surg Sports Traumatol Arthrosc. 2022;30(3):948–957. 10.1007/s00167-021-06473-3 PMC8901497 33582829

[8] Howell SM , Sappey‐Marinier E , Niesen AE , Nedopil AJ , Hull ML . Better Forgotten Joint Scores when the angle of the prosthetic trochlea is lateral to the quadriceps vector in kinematically aligned total knee arthroplasty. Knee Surg Sports Traumatol Arthrosc. 2023;31(12):5438–5445. 10.1007/s00167-023-07598-3 37792084

[9] Jeremic DV , Bellemans J , Sappey‐Marinier E , Howell SM , Hettwer W , Hull ML . Medial deviation of a 6° prosthetic trochlear groove after kinematically aligned total knee arthroplasty occurs in four types of Coronal Plane Alignment of the Knee (CPAK) and decreases the Forgotten Joint Score. Arthroplast Today. 2024;30:101569. 10.1016/j.artd.2024.101569 PMC11699096 39759179

[10] Keshmiri A , Maderbacher G , Baier C , Benditz A , Grifka J , Greimel F . Kinematic alignment in total knee arthroplasty leads to a better restoration of patellar kinematics compared to mechanic alignment. Knee Surg Sports Traumatol Arthrosc. 2019;27(5):1529–1534. 10.1007/s00167-018-5284-9 30421165

[11] Klasan A , Anelli‐Monti V , Putnis SE , Neri T , Kammerlander C . The effect of different alignment strategies on trochlear orientation after total knee arthroplasty. Knee Surg Sports Traumatol Arthrosc. 2024;32(7):1734–1742. 10.1002/ksa.12178 38606595

[12] Koutserimpas C , Saffarini M , Bonnin M , Hirschmann MT , Lustig S . Optimizing the patellofemoral compartment in total knee arthroplasty: is it time for dynamic assessment? Knee Surg Sports Traumatol Arthrosc. 2025;33(2):387–392. 10.1002/ksa.12450 39224026

[13] Meier M , Zingde S , Steinert A , Kurtz W , Koeck F , Beckmann J . What is the possible impact of high variability of distal femoral geometry on TKA? A CT data analysis of 24,042 knees. Clin Orthop Relat Res. 2019;477(3):561–570. 10.1097/CORR.0000000000000611 PMC6382181 30762689

[14] Nisar S , Palan J , Rivière C , Emerton M , Pandit H . Kinematic alignment in total knee arthroplasty. EFORT Open Rev. 2020;5(7):380–390. 10.1302/2058-5241.5.200010 PMC7407864 32818065

[15] Ollivier B , Luyckx T , Stragier B , Vandenneucker H . Kinematic alignment adequately restores trochlear anatomy, patellar kinematics and kinetics in total knee arthroplasty: a systematic review. Knee Surg Sports Traumatol Arthrosc. 2025;33(2):606–620. 10.1002/ksa.12401 39101252

[16] Orsi AD , Shatrov J , Plaskos C , Kreuzer S . Personalized alignment techniques better restore the native trochlear groove compared to systematic alignment techniques in total knee arthroplasty. Knee Surg Sports Traumatol Arthrosc. 2024;32(4):915–928. 10.1002/ksa.12104 38426570

[17] Rosa SB , Hazratwala K , Wilkinson MPR . Mismatch between trochlear coronal alignment of arthritic knees and currently available prosthesis: a morphological analysis of 4116 knees and 45 implant designs. Knee Surg Sports Traumatol Arthrosc. 2023;31(8):3116–3123. 10.1007/s00167-022-07251-5 PMC10356655 36456826

[18] Rossi R , Rosso F , Cottino U , Dettoni F , Bonasia DE , Bruzzone M . Total knee arthroplasty in the valgus knee. Int Orthop. 2014;38(2):273–283. 10.1007/s00264-013-2227-4 PMC3923939 24366186

[19] Shelton TJ , Gill M , Athwal G , Howell SM , Hull ML . Outcomes in patients with a calipered kinematically aligned TKA that already had a contralateral mechanically aligned TKA. J Knee Surg. 2021;34(1):87–93. 10.1055/s-0039-1693000 31288274

[20] Vendittoli P‐A , Martinov S , Blakeney WG . Restricted kinematic alignment, the fundamentals, and clinical applications. Front Surg. 2021;8:697020. 10.3389/fsurg.2021.697020 PMC8329359 34355018

[21] Vinograd O , Essa A , Steinberg N , Mitchnik IY , Avraham D , Rotem I , et al. Prospective comparison of short‐term outcomes in kinematic and mechanical alignment total knee arthroplasty. Clin Pract. 2025;15(9):162. 10.3390/clinpract15090162 PMC12468619 41002777

[22] Wang Q , Yan Z , Wang Q , Chen Y , Zhang X . The early outcome of robotic‐assisted functional versus mechanical alignment in total knee arthroplasty: a prospective randomized control trial. J Arthroplasty. 2025;40:S36–S44. 10.1016/j.arth.2025.08.030 40840662

